# The Role of Inflammatory Mediators in the Synergistic Toxicity of Ozone and 1-Nitronaphthalene in Rat Airways

**DOI:** 10.1289/ehp.8373

**Published:** 2006-06-22

**Authors:** Kara R. Schmelzer, Åsa M. Wheelock, Katja Dettmer, Dexter Morin, Bruce D. Hammock

**Affiliations:** 1 Department of Entomology; 2 Cancer Research Center and; 3 Department of Molecular Biosciences-School of Veterinary Medicine, University of California, Davis, California, USA

**Keywords:** 1-nitronaphthalene, arachidonic acid, cyclooxygenase, inflammatory mediators, linoleic acid, lipoxygenase, ozone

## Abstract

Ambient air is polluted with a mixture of pulmonary toxicants. Previous studies indicate that prior exposure to atmospheric oxidant pollutants such as ozone may significantly alter the response to other pollutants, such as 1-nitronaphthalene (1-NN). 1-NN, a component of the particulate exhaust from diesel engines, has been found at low concentrations in ambient air. Using a metabolomic approach, we investigated inflammatory responses in arachidonic and linoleic acid biochemical cascades (35 metabolites) and the expression of 19 cytokines/chemokines at three time points (2, 6, and 24 hr) following exposure to 1-NN with and without prior long-term O_3_ exposure. Long-term O_3_ exposure is associated with biochemical changes that have been shown to render the lung resistant to further O_3_ exposure. This study indicates that airways of O_3_-tolerant rats exhibited a low level of chronic inflammation, rendering the lungs more susceptible to other environmental pollutants such as 1-NN. Specifically, a 12.5-mg/kg dose of 1-NN to O_3_-tolerant rats produced significantly higher levels of cysteinyl-leukotrienes in bronchiolar lavage fluid even when compared to a 50-mg/kg dose of 1-NN in rats exposed to filtered air. Collectively, these results indicate that the combination of exposures as encountered in polluted ambient air are considerably more injurious to the lung than would be anticipated from previous studies employing single exposures. The observed synergism between O_3_ and 1-NN may be causally related to a shift in a T-helper 1 to T-helper 2 immune response in the airways.

Human epidemiologic studies have shown a relationship between ozone exposure and a decrease in pulmonary function in both children and adults (Balmes et al. 1997; Gauderman et al. 2004; Hiltermann et al. 1998). O_3_ is produced through photochemical reactions of volatile organic compounds in the presence of nitrogen oxides and sunlight. Exposure to O_3_ occurs primarily through inhalation, and causes injury and alterations to the respiratory tract. The National Ambient Air Quality Standard for O_3_ is set at 0.12 ppm [U.S. Environmental Protection Agency (EPA) 2000], which is often exceeded in urban areas during the summer months. Inhaled O_3_ first reacts with constituents of the epithelial lining fluid (e.g., micronutrient antioxidants such as ascorbate, urate, and vitamin E) and unsaturated lipids by reactive absorption.

O_3_ reaction with these components of the epithelial lining fluid produces reactive products including highly reactive aldehydes, hydrogen peroxide, superoxide, oxysterols, and hydroxyl radicals (Connor et al. 2004; Pulfer et al. 2005; Putman et al. 1997). Therefore, cellular responses to O_3_ are rarely a result of the direct reaction of O_3_ with cell surface components, but are rather mediated through a cascade of secondary products (Kelly et al. 1995; Pryor 1994). The lung’s inflammatory cells, such as macrophages, eosinophils, and neutrophils, constitute a major component of this cascade. Upon activation, these cells generate reactive oxygen and nitrogen species and cause substantial injury to lipid membranes, intra-cellular components, and proteins. In addition, O_3_ exposure activates epithelial cells and inflammatory cells to release cytokines, chemokines, and arachidonic acid. Arachidonic acid can be metabolized to leukotrienes (LTs), prostaglandins (PGs) hydroxyeicosatetraenoic acids (HETEs), dihydroxyeicosatetraenoic acids, and other metabolites.

Previous studies have examined acute O_3_ exposure as it relates to changes in eicosanoid levels and other cellular mediators, such as chemokines, cytokines, fibronectin and cell adhesion molecules (Driscoll et al. 1993; Ishii et al. 1997; Schlesinger et al. 1990). Furthermore, van Bree et al. (2001) studied the effects of exposure to 0.4 ppm O_3_ over 56 days in rats, and observed an acute inflammatory response characterized by an increase in neutrophils, protein, and albumin in bronchoalveolar lavage fluid (BALF) that reached a maximum 1 day after exposure. The acute inflammation resolved largely within 6 days during ongoing exposure, with neutrophils, protein, and albumin in BALF returning to control levels. However, the number of macrophages in BALF increased throughout the study. In addition, the collagen content in septa of the alveolar duct increased with ongoing exposure, marking the potential onset of fibrosis.

In the rat, long-term O_3_ exposure (> 90 days) leads to tolerance to further O_3_ exposure and remodeling of the distal airways, including an increase in nonciliated cell mass and volume fraction in terminal bronchioles and bronchiolarization of the alveolar duct (Plopper et al. 1994a, 1994b). Long-term O_3_ exposure has also been shown to cause increased sensitivity to the toxicity of the nitroaromatic 1-nitronapthalene (1-NN). Incomplete combustion of both gasoline and diesel fuel results in numerous nitrated polyaromatic hydrocarbons present in both the particulate- and gas-phase fractions of the exhaust (Draper 1986; Ishihara 2003). In addition, nitroaromatic compounds can be formed by nitration of the parent hydrocarbons through atmospheric reactions partially driven by the presence of O_3_ (Atkinson and Arey 1994).

Although 1-NN causes both centrilobular liver damage and bronchiolar epithelial damage in rats, the lung is the primary target tissue. At low and moderate doses, 1-NN causes an acute inflammatory event in the lungs that can be separated into two distinct temporal stages. The first stage occurs within the first 2 hr of exposure and is characterized by an increase in Clara cell toxicity. Clara cells contain high levels of cytochrome P450 monooxygenase 2B1, which is responsible for the bioactivation of 1-NN in the rat airway (Verschoyle et al. 1993). Reactive metabolites become bound covalently to cellular proteins; this process has been implicated in pulmonary epithelial cell injury associated with the high degree of P450 localization (Verschoyle et al. 1993; Wheelock et al. 2005). Ciliated cells, which contain only limited cytochrome P450 activity, are affected when adjacent to Clara cells. The second stage of 1-NN pulmonary inflammation occurs within 6 hr of exposure and is marked by infiltration of inflammatory cells into the interstitial areas and the onset of respiratory distress syndrome (at high doses). The infiltration of inflammatory cells into the lung is responsible for further damage to tracheal and bronchiolar epithelium. Sauer et al. (1997) correlated the respiratory distress syndrome induced by 1-NN with the infiltration of neutrophils, mononuclear cells, and small lymphocytes at both 6 and 24 hr after 1-NN exposure.

In this study we investigated the difference between the acute inflammatory responses produced by 1-NN in the airway lumen of rats following long-term O_3_ exposure versus that of controls exposed to filtered air. Moreover, our goal was to understand the roles of inflammatory mediators in the previously recognized synergistic toxicity of O_3_ and 1-NN. An examination of inflammatory mediators indicated both temporal- and dose-dependent effects, providing insights into alterations occurring at the cellular level in response to lung toxicants.

## Materials and Methods

### Chemicals

We obtained dextrose from Fisher Chemicals (Pittsburgh, PA), SeaPlaque low-melting-point agarose from FMC Bioproducts (Rockland, ME), and ultrapure urea from US Biological Corp. (Cleveland, Ohio). Protease inhibitor cocktail III was acquired from CalBioChem (LaJolla, CA). The oxylipins were obtained from Cayman Chemical (Ann Arbor, MI), or synthesized in-house (Kim et al. 2004; Newman et al. 2002; Watanabe et al. 2003). We purchased solid phase extraction (SPE) cartridges (Oasis HLB 60 mg) from Waters (Milford, MA) and ethyl acetate, phosphoric acid, and glacial acetic acid of HPLC grade or better from Fisher Scientific (Pittsburgh, PA). Omni-Solv acetonitrile and methanol purchased from EM Science (Gibbstown, NJ) were used for all reverse-phase HPLC analyses. All other chemical reagents were purchase from Sigma Chemical Company (St. Louis, MO). 1-NN was synthesized as previously described (Crivello 1981).

### Animal exposures

Male Sprague-Dawley rats were purchased from Harlan (Indianapolis, IN). All protocols and procedures were approved by the University of California Davis Animal Care and Use Committee and are in accordance with the National Institutes of Health *Guide for the Care and Use of Laboratory Animals* (Institute of Laboratory Animal Resources 1996) to ensure that the animals were treated humanely with regard for alleviation of suffering. Animals were fed *ad libitum* and housed in HEPA-filtered cage racks at the University of California, Davis, in a facility accredited by the Association for Assessment and Accreditation of Laboratory Animal Care. Animals were held for at least 5 days before use in an experiment. Seventy-two rats (281–318 g body weight, 8–10 weeks of age) were randomly assigned to 18 groups (*n* = 4) (Table 1).

#### Filtered air versus O_3_

Groups 1–9 were exposed to filtered air for 90 days and groups 10–18 were exposed to 0.8 ppm O_3_ 8 hr/day for 90 days, as previously described (Paige et al. 2000). On the 91st day all rats were returned to ambient air, and the rats in groups 1–3 and 10–12 received a single intraperitoneal (ip) injection of corn oil (vehicle).

#### Dose and temporal response

Groups 4–6 and 13–15 received an ip injection of 12.5 mg/kg 1-NN in corn oil. Groups 7–9 and 16–18 received an ip injection of 50 mg/kg 1-NN in corn oil. Groups 1, 4, 7, 10, 13, and 16 were sacrificed 2 hr postinjection, groups 2, 5, 8, 11, 14 and 17 were sacrificed 6 hr postinjection, and groups 3, 6, 9, 12, 15, and 18 were sacrificed 24 hr postinjection. Animals were sacrificed by a lethal dose of pentobarbital. Time intervals were selected to observe initial inflammatory phase, injury phase, and repair phase. We collected bronchiolar lavage fluid (BLF) immediately after euthanasia, using the lysis-lavage method as described by Wheelock et al. (2004). Briefly, the alveolar region was sealed off through partial inflation with agarose. The airways were then lavaged with an isotonic dextrose solution. The collected sample thus differs from the more commonly used BALF, where both airways and alveoli are lavaged.

### Oxylipin analysis

#### On-line SPE extraction

Analysis of cysteinyl leukotrienes (cys-LT) was performed by online solid phase extraction coupled to HPLC-tandem mass spectrometry (MS). BLF samples were diluted 1:1 with 2.5 mM phosphoric acid (pH 3.8), and spiked with internal standard [12-(3-cyclohexyl-ureido)-dodecanoic acid, 27.9 nM]. Twenty microliters of each sample was injected on the Oasis HLB pre-column (20 mm × 2.1 mm; Waters), which was then washed using aqueous solvent to remove interfering matrix components such as salts and proteins. Following the injection and wash steps, the pre-column was eluted in the reverse direction onto the analytical HPLC column. Analytes were separated on a reversed-phase HPLC column [Luna 5 μm C18 (2), 150 × 2.0 mm; Phenomenex, Torrance, CA] using gradient elution. Solvent A consisted of 8.3 mM acetic acid, with the pH adjusted to 5.7 with ammonium hydroxide; solvent B was acetonitrile: methanol:acetic acid (65:35:0.1), and the flow rate was 200 μL/min. The solvent gradient started with 99% solvent A and 1% solvent B; it was held for 2.2 min, then increased to 30% solvent B and held for 4 min and further increased to 50% solvent B for 6.5 min. Finally, the column was washed with 100% solvent B for 2 min, then returned to the starting conditions and equilibrated for 1.5 min before the next injection.

#### Off-line SPE extraction

BLF aliquots (250 μL) were diluted 1:1 vol/vol with 2.5 mM phosphoric acid immediately before SPE extraction. Surrogates containing 26.7 nM of 6-keto prostaglandin F_1α_–d_4_ (PGF_1α_–d_4_), 10(11)-epoxyheptadecanoic acid, and 10,11-dihydroxynonadecanoic acid were added to each sample before SPE was performed. Oasis-HLB 60 mg cartridges (Waters) were preconditioned with 2 mL of methanol and 2 mL of 2.5 mM phosphoric acid (pH 3.8). After the samples were applied, the cartridges were washed with 2 mL of 2.5 mM phosphoric acid (pH 3.8). The analytes were eluted with 2 mL ethyl acetate; all elutions were performed by gravity. The ethyl acetate extract was evaporated under nitrogen, and the samples were resuspended in 100 μL methanol containing 26.7 nM of the internal standards (1-phenyl-3-hexanoic acid urea and 1-cyclohexyl-dodecanoic acid urea). Samples were vortexed for 5 min, transferred to autosampler vials with low volume inserts and stored at −20°C until analysis.

#### LC/MS/MS method

Liquid chromatography/tandem MS (LC/MS/MS) analysis of oxylipins was performed as previously described (Schmelzer et al. 2005). We used a Waters 2790 separation module equipped with a 2.0 × 150 mm, 5 μm Luna C18 (2) column (Phenomenex), and the column temperature was held at 40°C. The samples were kept at 10°C in the autosampler, and 10 μL of each sample was injected. The solvent flow rate was fixed at 350 μL/min. The solvent gradient started with 85% water with 0.1% glacial acetic acid (solvent A) and 15% acetonitrile: methanol:acetic acid (88:12:0.1) (solvent B) and was held for 30 sec. Solvent B was then increased to 30% by 2 min, then to 55% by 8 min, and 75% by 28 min. Finally, the column was washed with 100% solvent B for 5 min, then returned to the starting conditions and equilibrated for 5 min before the next injection. A Quattro Ultima tandem-quadrupole mass spectrometer (Micromass, Manchester, UK) equipped with an electrospray ionization source was used in negative ion mode. Compounds were analyzed in multiple reaction monitoring mode and quantified by using surrogate corrected calibration curves. Calibration curves were generated by 10 μL injections of seven different calibration standards containing each analyte, surrogate, and internal standard. For online SPE extraction, the ionization source was used in positive ion mode, which allowed for greater sensitivity for the cys-LTs. The cys-LT method, which includes LTC_4_, LTD_4_, LTE_4_, LTF_4_, and *N*-acetyl LTE_4_, was modified from the method of Kishi et al. (2001).

### Cytokine and chemokine analysis

We purchased rat protein cytokine/chemokine-array kits from RayBiotech (Norcross, GA). Cytokine/chemokine analysis was performed using BLF from a pooled sample (*n* = 4) from animals that received filtered air or O_3_ without 1-NN, because no induction of cytokines or chemokines was expected. BLF from rats that received 1-NN were analyzed with individual arrays. Briefly, the membranes were blocked with a blocking buffer; then 1 mL BLF from each treatment group was added to the membranes, and incubated at room temperature for an additional 2 hr. The membranes were washed and incubated with 1 mL primary biotin-conjugated antibody at room temperature for 2 hr, followed by 2 mL horseradish peroxidase–conjugated streptavidin at room temperature for 30 min. The membranes were developed using ECL Plus solution (Amersham, Piscataway, NJ) and were exposed to Storm imager (Chemi-fluorescent detection), and signals were analyzed using ImageQuant 5.1 software (Molecular Dynamics, Sunnyvale, CA). These arrays are semiquantitative; therefore, the individual cytoines/chemokines had to be present in 75% of the samples to be considered an increase.

### Statistical analysis

We used the Student’s *t*-test to determine differences between exposed groups and control groups. Values with *p* < 0.01 were considered significant. For statistical analysis, analytes below the limit of detection were assigned a value at the limit of detection.

## Results and Discussion

The long-term inhalation of O_3_ fundamentally alters the cellular homeostasis of the respiratory system (Pinkerton et al. 1993). This change is evident with a subsequent exposure to another prevalent environmental contaminant, 1-NN; previous pathology studies have shown that O_3_-tolerant animals exhibit a dose-dependent increased susceptibility to 1-NN (Paige et al. 2000a, 2000b). The observed synergistic effects were localized to the central acinus, an area that undergoes distinct biochemical and structural changes in response to long-term O_3_ exposure. Specifically, 24 hr after 1-NN administration, 17% of the distal airway epithelium was denuded and 8% of the remaining cells were necrotic in O_3_-exposed animals at the lowest 1-NN dose tested (50 mg/kg). In the corresponding filtered air-exposed animals, the distal airway epithelium remained continuous and only 6% of the cells were necrotic (Paige et al. 2000b). At higher 1-NN doses, the synergistic effects were even more pronounced (Paige et al. 2000b). Although the increased toxicity to 1-NN can be explained in part by an induced expression of P450 2B1 in O_3_-tolerant animals (Paige et al. 2000a) with subsequent alterations in the protein adduct formation pattern (Wheelock et al. 2005), the mechanisms underlying the synergistic toxicity are largely unknown. The present study was designed to investigate the occurrence and importance of alterations in the inflammatory response to 1-NN in O_3_-tolerant animals. We found that rats exposed to O_3_ and 1-NN produce inflammatory mediators analogous to an allergic response, whereas rats exposed to filtered air and 1-NN produce inflammatory mediators consistent with an acute inflammatory response.

### O_3_ versus filtered air (no 1-NN exposure)

**Figure 1:**
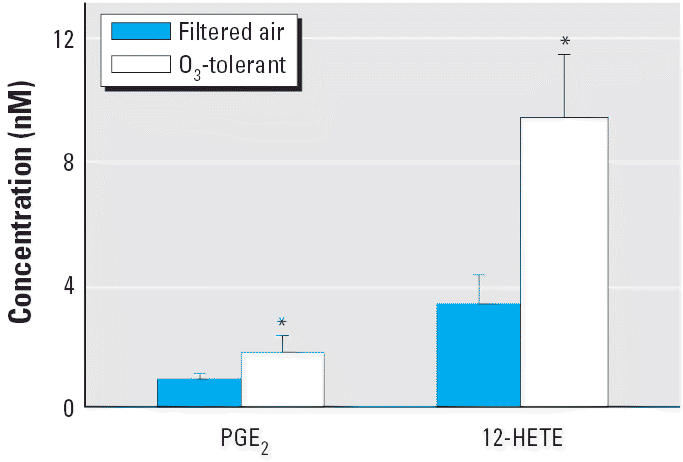
Differences in the metabolites PGE_2_ and 12-HETE in rats exposed to O_3_ or filtered air for 90 days (mean ± SD; *n* = 12). *Statistically significant (*p* < 0.01.

The rat respiratory system is much less sensitive to O_3_ than that of nonhuman primates, and exposures to concentration > 0.5 ppm are required to mimic the pathologic effects on the primate lung at ambient O_3_ concentrations (Barr et al. 1990). To reflect this difference, we intentionally set the level of O_3_ exposure in this study (0.8 ppm) to about twice as high as typical peak values in polluted urban areas. The levels of PGE_2_ and 12-HETE were significantly higher in BLF from O_3_-tolerant rats compared with BLF from filtered air-exposed rats (Figure 1). PGE_2_ is a potent vasodilator that can enhance edema and leukocyte emigration. Previous studies have shown that chronic O_3_ exposure leads to an increased number of Clara cells in the central acinus, which can produce both PGE_2_ and 12-HETE (Van Scott et al. 1990). Additionally, Marom et al. (1983) found that 12-HETE, in nanomolar concentrations, is capable of stimulating the release of mucosal proteins from human airways. In many airway diseases, mucus overproduction and cell metaplasia are two clinical hallmarks that are frequently associated with airway obstruction and inflammation. Animal experiments with O_3_ exposure demonstrated an increase of mucous cell hyperplasia in the airway epithelium (Paige and Plopper 1999). In the present study, we found no significant differences in cytokine production between filtered air- and O_3_-exposed rats (data not shown).

### 1-NN temporal and dose response in filtered air-exposed rats

In polluted urban areas, we are exposed to low levels of 1-NN through inhalation of both the aerosol and particulates. Because no satisfactory regimen for inhalation exposure to 1-NN currently is available, the toxicant was administered through ip injection in this study, as well as in the preceding pathology studies (Paige and Plopper 1999; Paige et al. 2006b). No studies correlating doses through different administration routes have been published for 1-NN. However, corresponding studies for the parent compound naphthalene, which has a similar mechanism of toxicity, correlated injury levels in distal airways following inhalation of current Occupational Safety and Health Administration standards (10 ppm) with ip administration of 100 mg/kg in mice, approximately 25% of the median lethal dose (LD_50_) (Warren et al. 1982; West et al. 2001). In addition, we recently showed that 1-NN is activated in the airway epithelium before hepatic activation (also following ip administration), and injury to the airway epithelium is likely independent of hepatic metabolism (Wheelock et al. 2005). In that study, we used two doses: a low dose of 12.5 mg/kg to mimic an environmentally relevant dose and a high dose of 50 mg/kg, approximately 36% of the LD_50_ (Verschoyle and Dinsdale 1990), to match previous pathology studies. Also, we studied three different time points.

**Figure 2:**
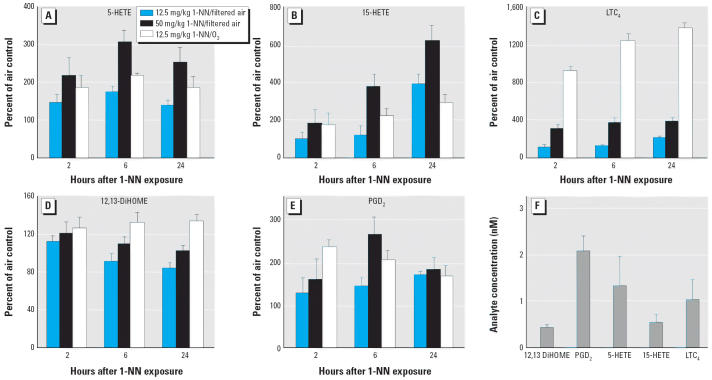
1-NN exposure produced temporal changes (mean ± SD; *n* = 4) in BLF oxylipins derived from epoxygenase, cyclooxygenase (COX), and lipoxygenase (LOX) pathways. All values in A–C are depicted as the percent of control rats receiving filtered air and vehicle without 1-NN. (*A–C*) LOX-dependent metabolites 5-HETE (*A*), 15-HETE (*B*), and LTC_4_ (*C*). (*D*) Epoxygenase-dependent metabolite 12,13-DiHOME. (*E*) COX-dependent metabolite PGD_2_. (*F*) Analyte concentrations in rats exposed to filtered air and treated with vehicle.

The profile of lipid mediators and cytokines changed at each time point denoting the inflammatory stages (i.e., injury and repair). Figure 2 depicts five oxylipin metabolites at the three sampling times, 2, 6, and 24 hr. The temporal profile for the linoleic diols [dihydroxyoctadecenoic acid (DiHOMEs)] indicates that the greatest increase occured at 2 hr; then the concentration slowly decreased throughout the study. A similar pattern indicating injury and repair phases was displayed by 5-HETE, but with the maximum occurring at 6 hr. The 15-HETE concentration increases over time for both the 12.5 and 50 mg/kg doses, indicating that 15-HETE may be involved with cellular proliferation and repair phases (Kuhn 1996). PGD_2_ concentrations reached a maximum at 6 hr postinjection, and had decreased again after 24 hr, with a statistically significant change in the high-dose animals (50 mg/kg) but not in the low-dose animals (12.5 mg/kg). Finally, LTC_4_ constantly increased throughout the three time points, indicating that it may be responsible for the bronchoconstriction that occurs during the early and late inflammatory responses. In most instances, a dose-dependent effect in expression levels was observed when the dose of 1-NN was increased from 12.5 to 50 mg/kg.

Additionally, at a 1-NN dose of 12.5 mg/kg, we detected the cytokines tumor necrosis factor-α (TNF-α) and interleukin-1α (IL-1α) only at 2 hr postinjection, whereas IL-6 and IL-10 were present at 6 and 24 hr post-injection (Supplemental Material, Table S1A, available online at http://www.ehponline.org/docs/2006/8373/suppl.pdf). Increasing the 1-NN dose to 50 mg/kg again increased the inflammatory response, but with similar profiles. TNF-α and IL-1β were detected at 2 hr, along with cytokine-induced neutrophil chemoattractant-1 (CINC-2), granulocyte-macrophage colony-stimulating factor (GM-CSF), macrophage inflammatory protein-3α, IL-6, leptin, monocyte chemoattractant protein-1, ciliary neuronotrophic factor (CNTF), interferon-γ (IFN-γ), and IL-10. Data from both doses of 1-NN indicate an acute inflammatory event, with the 50 mg/kg dose producing more cellular injury. As time progressed, the number of immunostimulatory cytokines, such as TNF-α and IL-1, decreased. TNF-α and IL-1 play a fundamental role in initiating the inflammatory cascade. Specifically, they contribute to polymorpho-nuclear cell maturation, trafficking, and activation. As time progresses, the number of immunoregulatory cytokines increases, which counteracts various aspects of inflammation. For example, IL-10 balances this amplification by promoting the mRNA degradation of IL-1, IL-6, and TNF-α (Howard 1992; Malefyt 1991).

Overall, the results of the present study were consistent with the pathologic responses to 1-NN observed in previous studies (Paige et al. 1997; Sauer et al. 1997). Moreover, the sensitivity of the inflammatory mediator analysis allowed the detection of these effects at exposure levels as low as 12.5 mg/kg. In contrast, animals exposed to even 25 mg 1-NN/kg are pathologically indistinguishable from control animals (Paige et al. 1997).

### Synergistic effects of O_3_ and 1-NN (12.5 mg/kg)

Previous studies have shown that long-term O_3_ exposure causes cellular remodeling of the respiratory tract (Chang et al. 1992; Harkema et al. 1987; Paige and Plopper 1999; Stockstill et al. 1995). In the terminal bronchioles, ciliated and Clara cells begin invading the alveolar airspace. Additionally, there is evidence of bronchiolar metaplasia of the alveolar ducts (Pinkerton et al. 1993), epithelial inflammation, and interstitial fibrosis. Other cellular changes include proliferation of type II pneumocytes (Chang et al. 1992), fibrosis (Stockstill et al. 1995), interstitial thickening in the central acinus, and an increase in alveolar macrophages (Paige and Plopper 1999). Although this adaptation makes the lung more resistant to further O_3_ insult, O_3_-tolerant lungs are more susceptible to secondary lung irritants such as 1-NN (Paige et al. 2000a, 2000b). The results of the present study indicate that the observed synergistic toxicity is correlated with an altered inflammatory response of the airway. The 12.5 mg/kg dose of 1-NN with prior O_3_ exposure led to a greater inflammatory response than the same dose of 1-NN and filtered air-exposure. Thirteen oxylipins and 11 cytokines were statistically different in response to 1-NN and O_3_ as compared with 1-NN and filtered air (Supplemental Material, Table S1A,B; available online at http://www.ehponline.org/docs/2006/8373/suppl.pdf).

DiHOMEs, 15-HETE, 11-HETE, 12-HETE, 5-HETE, 6-keto- PGF_1α_, throm-boxane B_2_, PGE_2_, PGD_2_, and LTB_4_ are statistically higher in O_3_-tolerant rats exposed to 12.5 mg/kg 1-NN, than in filtered air–exposed animals receiving the same 1-NN dose (Supplemental Material, Table S1B; available online at http://www.ehponline.org/docs/2006/8373/suppl.pdf). Figure 2 shows comparisons of O_3_-exposed rats (dose 12.5 mg/kg 1-NN) to filtered air-exposed rats (both 12.5 and 50 mg/kg 1-NN doses). In contrast to filtered air-exposed rats, DiHOME concentrations in BLF did not decrease with time but remained elevated until the end of the experiment. Sustained micromolar concentrations of DiHOME have been observed in patients under inflammatory and hypoxic conditions (Dudda 1996; Ozawa et al. 1988) and cardio-pulmonary toxicity (Hayakawa et al. 1990; Ishizaki 1996; Ozawa et al. 1988). Similar temporal patterns were seen for 5-HETE, which is rapidly produced upon inflammatory insult; 5-HETE is a potent mediator of neutrophil function, with chemotactic activity and the ability to modulate lysosomal enzyme release (Spanbroek et al. 2000). At 2 hr after 1-NN administration, the 15-HETE concentration was significantly higher in O_3_-tolerant rats than in filtered air-exposed rats; by 24 hr, the 15-HETE level was significantly lower than in the filtered air-exposed rats administered 1-NN. Bronchial mucosal biopsies from asthma patients are known to have increased levels of 15-lipoxygenase, the enzyme that produces 15-HETE (Bradding 1995). In the present study, O_3_-tolerant rats given 1-NN showed an initial increase in PGD_2_ concentration, but by 24 hr the PGD_2_ concentrations were no longer statistically different from filtered air-exposed rats administered 1-NN. The major source of PGD_2_ is activated mast cells, which contribute significantly to bronchoconstriction, airway hyperreactivity, and inflammation (Kleeberger et al. 2001). The most striking differences are seen in the cys-LTs at 2, 6, and 24 hr (Figure 2 and Supplemental Material, Table S1B; available online at http://www.ehponline.org/docs/2006/8373/suppl.pdf). The cys-LTs contract smooth muscle of the airway (Barnes et al. 1984), act on the vasculature to produce vasodilation, and increase vascular permeability (Drazen et al. 1980). Additionally, cys-LTs stimulate mucous secretion and interfere with mucociliary clearance (Marom et al. 1982). It is widely believed that cys-LTs are important mediators in bronchial asthma. Furthermore, increased LTB_4_ and cys-LT levels have been found in human BALF after O_3_ exposure (Coffey et al. 1996). Sources of cys-LT production include eosinophils, mast cells, and macrophages. Under certain conditions, mast cells produce and release IL-4, which plays an important role in the differentiation and maturation of T cells towards the T helper (T_H_) 2 phenotype.

**Figure 3:**
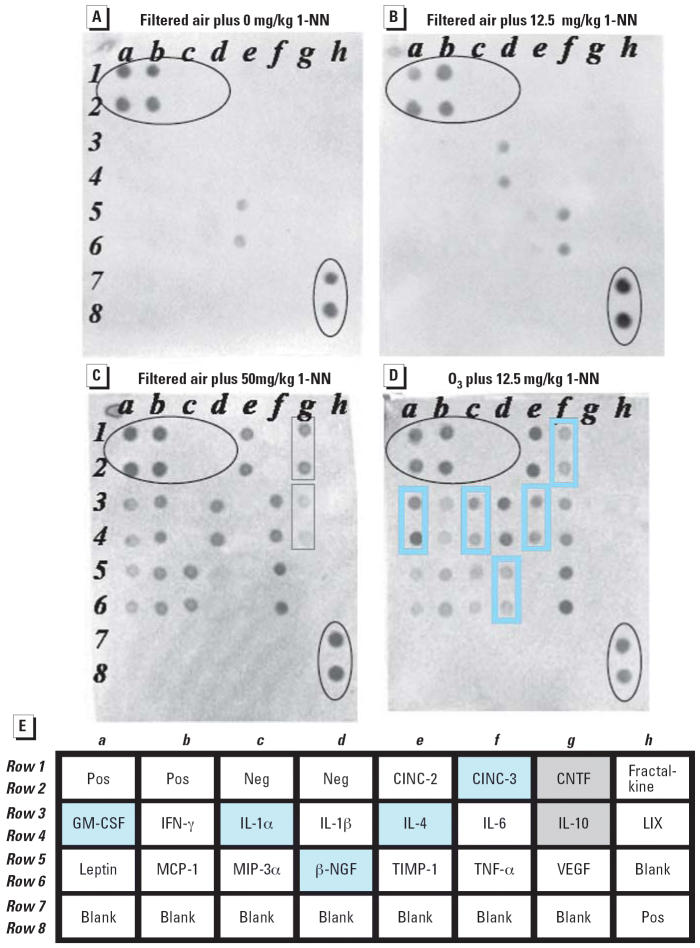
BLF chemokine/cytokine profiles of rats exposed to different doses of 1-NN ± O_3_ at the 2-hr time point. Abbreviations: LIX, LPS inducing factor; MCP, monocyte chemoattractant protein; MIP, macrophage inflammatory protein; TIMP, tissue inhibitor of metalloproteinases; VEGF, vascular endothelial growth factor. Circled regions in the upper left and lower right portions of the arrays indicate positive and negative controls, whereas the rectangles indicate cytokines that showed selective responses to 1-NN ± O_3_. (*A*) Control rats (*n* = 4, pooled sample; exposure group 1, filtered air without 1-NN). (*B*) Individual rat (exposure group 4, filtered air and 12.5 mg/kg 1-NN). (*C*) Individual rat (exposure group 7, filtered air and 50 mg/kg 1-NN). (*D*) Individual rat (exposure group 13, O_3_-tolerant rats exposed to 12.5 mg/kg 1-NN). Analysis of the arrays revealed an increase in levels of certain cytokines and chemokines in response to different concentrations of 1-NN (*B,C*). An individual cytokine/chemokine had to be present in 75% of the samples to be considered increased; for example IFN-γ is visable in (*D*) but was not present in 75% of the samples, and is therefore not considered increased above control. Also, a different set of cytokines/chemokines are present in (*D*). (*A–D*) are representative of multiple experiments, all of which gave similar results. (*E*) Array map for the panel of 19 secreted cytokines/chemokines from Ray Biotec. In (*C*) and (*D*), the boxes indicate the cytokines and chemokines that differ in response to 50 mg/kg 1-NN plus filtered air exposure (gray boxes in *C* and *E*) or 12.5 mg/kg 1-NN plus O_3_ exposure (blue boxes in *D* and *E*), No statistical significance was found in animal weight or in BLF protein concentration among the various groups (*p* < 0.05).

The synergistic effects of O_3_ and 1-NN were most apparent when examining the cytokine/chemokine arrays 2 hr postexposure (Figure 3). In comparison, at 6 hr post-exposure, no pathologic signs of synergistic toxicity can be observed with 50 mg/kg 1-NN (Lee MG, personal communication). Figure 3A shows the absence of inflammatory cytokines in control rats exposed to filtered air and vehicle. Figure 3B indicates the presence of TNF-α, and IL-1β in a rat exposed to filtered air and 12.5 mg/kg 1-NN. Figure 3C depicts the presence of cytokines/chemokines—including IL-10 and CNTF, which are immunoregulatory and counteract various aspects of inflammation—in a rat exposed to filtered air and 50 mg/kg 1-NN. Figure 3D depicts immunostimulatory mediators including β-nerve growth factor (β-NGF), IL-1α, CINC-3, and IL-4 and an increase in GM-CSF in a rat exposed to O_3_ and 12.5 mg/kg 1-NN. Moreover, there are no immunoregulatory cytokines/chemokines present in Figure 3D. The abundance of seven cytokines/chemokines were different in O_3_-tolerant rats administered 12.5 mg/kg 1-NN compared with rats exposed to filtered air and 50 mg/kg 1-NN. The O_3_-tolerant rats produced a unique cytokine profile that is comparable with an influx of T_H_2 cells and eosinophils and the development of airway hyperresponsiveness. The T_H_2 inflammatory event is characterized by an increase in IL-4, IL-5, GM-CSF, and β-NGF (Bosson et al. 2003; Braun et al. 1998; Driscoll et al. 1993; Ishii et al. 1997; Maggi et al. 1992; Sanico et al. 2000; Spanbroek et al. 2000; Stankova et al. 1995). IL-4 stimulates B cells to produce antigen-specific IgE and inhibits the production of T_H_1 cytokines such as IFN-γ (Maggi et al. 1992). IFN-γ was not detected in the O_3_-tolerant rats, but it was detected in filtered air-exposed rats given 50 mg/kg 1-NN. Although we did not measure IL-5, previous work has shown that O_3_ increases the amount of IL-5 and GM-CSF (Joad et al. 1994). These proteins promote the recruitment, activation, and survival of eosinophils. Activated eosinophils are primed for the enhanced production of cys-LT, which may be a major source of the hyperresponsiveness. Finally, β-NGF alters sensory nerve function and promotes allergic inflammation, bronchial hyperactivity, and airway obstruction (Quarcoo et al. 2004).

The ability of O_3_ to enhance allergic sensitization in mice has been well documented. Exposures in mice of O_3_ > 0.13 ppm showed greater anaphylactic sensitivity to intravenous challenge with ovalbumin (Gershwin et al. 1981). Increases in mediators important in inflammation, such as IL-6, IL-5, GM-CSF, and PGE_2_, have also been documented in BALF recovered from humans exposed to O_3_ (Bosson et al. 2003; Devlin et al. 1996; Joad et al. 1994). Our results in the present study illustrate that the interaction between O_3_ and 1-NN alters the inflammatory response in a way that would not have been predicted from individual toxicologic profiles. Tolerance to O_3_ is associated with increased activity of several antioxidant enzymes (Plopper et al. 1994) and elevated levels of glutathione (Duan et al. 1996). Although the electrophilic 1-NN metabolites supposedly are detoxified by glutathione conjugation (Watt et al. 1999), this reaction does not impart total resistance to 1-NN (Paige et al. 2000a, 2000b). Our data suggest that O_3_-tolerant rats produce an allergic inflammatory response upon exposure to 1-NN, which may explain the mechanisms underlying the unexpected enhanced sensitivity to 1-NN. The mechanism by which O_3_ remodeling establishes the T_H_2-mediated allergic inflammation is unknown. This might result from the direct effect of O_3_ on increasing membrane permeability, allowing toxicants to penetrate deeper into tissue or allowing more leukocytes into the lungs. Additionally, O_3_ may modulate the local cytokine milieu within the airways through the enhanced recruitment of leukocytes into the lung, such as mast cells and eosinophils, which produce IL-4. Alternatively, O_3_ may also have a direct effect on the process of allergic sensitization by modifying proteins in antigen-presenting cells that function within airways.

## Conclusion

In this study we examined mechanisms underlying the synergistic toxicity between O_3_ and 1-NN by analyzing the differences in inflammatory profiles. The inflammatory profile consisted of 35 oxylipin metabolites and 19 different cytokines. Filtered air and O_3_-tolerant rats were exposed to 1-NN at three different doses (0, 12.5, and 50 mg/kg), and BLF was collected at three different time points. The present study demonstrates a sensitive method to detect differences in inflammatory responses and provides further evidence that O_3_ adaptation produces an allergen-like immune response to 1-NN even at relatively low doses (12.5 mg/kg). Further investigations are now required to determine whether similar profiles can be found in human patients exposed to such co-pollutants.

The synergism of O_3_ and 1-NN is probably due to the remodeling that occurs after long-term O_3_ exposure. This remodeling appears to establish a T_H_2 inflammatory response rather than a T_H_1 response seen in filtered air-exposed controls. The alteration in cytokine/chemokine data, along with the high concentration of cys-LT, provides strong support for this argument. BLF from rats exposed to O_3_ and 12.5 mg/kg 1-NN contained IL-4, GM-CSF, β-NGF, TNF-α, IL-1, and a high concentration of LTC_4_ (923% of control). The same dose administered to filtered air-exposed rats produces only increases in TNF-α, IL-1α, and LTC_4_ (120% of control).

Currently, ambient air quality standards (U.S. EPA 2000) are based on the toxicologic effects of a single pollutant (e.g., O_3_). Consequently, the health risk of breathing a mixture of pollutants may have been underestimated. Although extrapolating such experimental data to human exposures should be done with great caution, our findings suggest that cellular and biochemical changes associated with chronic oxidant stress, frequently encountered in highly polluted areas, may significantly elevate susceptibility to some co-pollutants, thereby posing a greater risk to human health.

## Figures and Tables

**Table 1:** Exposure regimen of rats by group number.

	Filtered air			O_3_		
1-NN (mg/kg)	2 hr	6 hr	24 hr	2 hr	6 hr	24 hr
0 (vehicle)	1	2	3	10	11	12
12.5	4	5	6	13	14	15
50	7	8	9	16	17	18

Rats were sacrificed at 2, 6, or 24 hr postinjection; *n* = 4 rats/group.
