# Supplementary material for: The Role of Inflammatory Mediators in the Synergistic Toxicity of Ozone and 1-Nitronaphthalene in Rat Airways
Source: Environ Health Perspect. 2006 Jun 22;114(9):1354–60. doi: 10.1289/ehp.8373 (PMC1570074; doi:10.1289/ehp.8373)
Supplement: Supplemental Figures and Tables [file ehp0114-001354s1.pdf]

Table S1A. Time and Dose Response in Inflammatory Cytokines and Chemokines

	12.5 mg/kg 1-NN + Air			12.5 mg/kg 1-NN + Ozone			50 mg/kg 1-NN + Air			50 mg/kg 1-NN + Ozone		
**Mediators**	**2 hrs**	**6 hrs**	**24 hrs**	**2 hrs**	**6 hrs**	**24 hrs**	**2 hrs**	**6 hrs**	**24 hrs**	**2 hrs**	**6 hrs**	**24 hrs**
**TNFα**	+	-	-	+	-	-	+	-	-	++	+	-
**IL-1α**	+	-	-	+	-	-	-	+	-	+	-	-
**IL-1β**	-	-	-	++	+/-	-	+	-	-	+++	+	-
**IFN-γ**	-	-	-	+	-	-	++	+	-	+	+	+/-
**IL-6**	-	+	+	+	+	+	+	+	+	+	+	+
**IL-10**	-	+	+	-	-	-	+	++	+	-	-	-
**CNTF**	-	-	-	-	-	-	+	+	+	-	-	-
**Leptin**	-	-	-	+	+/-	-	+	+	-	+	+	-
**MIP-3α**	-	-	-	+/-	+/-	-	+	+	+/-	+	+/-	+/-
**MCP-1**	-	-	-	+/-	+	+	+	++	+	+	++	++
**CINC-2**	-	-	-	++	++	+	+	-	-	++	++	+
**CINC-3**	-	-	-	+	+	-	-	-	-	+	+	+
**GM_CSF**	-	-	-	++	++	++	+	+	-	+++	+++	++
**IL-4**	-	-	-	+	+	+	-	-	-	+	+	+
**β-NGF**	-	-	-	+	+	+	-	-	-	+	+	+

- = non detected in 75% of the samples
+/- = present in 75% of the samples
+ = present in all samples but lower than the membrane's positive control
++ = present in all samples and equal to membrane's positive control
+++ = present in all samples and greater than membrane's positive control
Black background box represents the immunostimulatory cytokines
Grey lettering represents immunosuppressive cytokines.
Gray background box represents chemokines that are indicative of a T_H2- allergic response

B. Time and Dose Response in Inflammatory Oxylipins

	12.5 mg/kg 1-NN + Air			12.5 mg/kg 1-NN + Ozone			50 mg/kg 1-NN + Air			50 mg/kg 1-NN + Ozone		
**Lipid Mediators**	**2 hrs**	**6 hrs**	**24 hrs**	**2 hrs**	**6 hrs**	**24 hrs**	**2 hrs**	**6 hrs**	**24 hrs**	**2 hrs**	**6 hrs**	**24 hrs**
**9,12,13-TriHOME**	39 ± 5	70 ± 6	67 ± 5	50 ± 4	84 ± 6	77 ± 3	59 ± 23	91 ± 16	105 ± 21	85 ± 20	80 ± 16	93 ± 14
**9,10,13-TriHOME**	77 ± 9	102 ± 5	133 ± 10	99 ± 7	110 ± 2	145 ± 6	76 ± 22	109 ± 20	135 ± 19	91 ± 10	107 ± 10	140 ± 13
**12,13-DiHOME**	112 ± 8	93 ± 13	84 ± 10	127 ± 18	133 ± 12	135 ± 8	122 ± 10	111 ± 22	104 ± 29	126 ± 37	148 ± 21	159 ± 27
**9,10-DiHOME**	102 ± 6	103 ± 7	104 ± 5	125 ± 12	125 ± 10	124 ± 7	128 ± 11	116 ± 6	109 ± 5	134 ± 14	133 ± 10	132 ± 15
**15-HETE**	96 ± 40	123 ± 42	391 ± 53	177 ± 56	220 ± 45	291 ± 37	183 ± 52	376 ± 68	617 ± 83	278 ± 77	390 ± 60	421 ± 58
**11-HETE**	156 ± 50	157 ± 42	205 ± 26	265 ± 48	250 ± 34	193 ± 15	259 ± 40	500 ± 28	221 ± 48	329 ± 51	436 ± 49	210 ± 36
**12-HETE**	116 ± 26	137 ± 41	209 ± 25	317 ± 54	325 ± 63	368 ± 31	264 ± 101	532 ± 111	450 ± 106	497 ± 92	484 ± 39	712 ± 95
**5-HETE**	145 ± 22	173 ± 16	138 ± 14	188 ± 29	218 ± 6	184 ± 31	218 ± 44	308 ± 32	252 ± 42	248 ± 38	374 ± 48	292 ± 58
**6-keto-$PGF_{1α}$**	92 ± 11	93 ± 11	119 ± 30	119 ± 15	129 ± 24	127 ± 36	133 ± 24	152 ± 33	121 ± 9	140 ± 17	163 ± 26	127 ± 21
**TXB_2**	92 ± 5	124 ± 23	81 ± 4	102 ± 9	165 ± 16	104 ± 18	149 ± 15	158 ± 32	184 ± 20	211 ± 55	225 ± 24	187 ± 42
**PGE_2**	173 ± 31	209 ± 21	143 ± 21	267 ± 36	269 ± 10	175 ± 46	414 ± 69	541 ± 46	364 ± 62	523 ± 29	731 ± 52	386 ± 34
**PGD_2**	130 ± 34	143 ± 21	169 ± 11	235 ± 16	204 ± 22	165 ± 27	159 ± 49	263 ± 41	181 ± 31	233 ± 23	334 ± 36	120 ± 58
**LTB_4**	141 ± 20	121 ± 12	114 ± 12	177 ± 17	132 ± 9	120 ± 10	257 ± 41	153 ± 28	120 ± 10	342 ± 33	188 ± 48	120 ± 0
**LTC_4**	120 ± 19	133 ± 21	216 ± 13	923 ± 50	1234 ± 74	1368 ± 61	318 ± 33	375 ± 55	385 ± 50	975 ± 87	1359 ± 74	NV
**LTD_4**	104 ± 11	124 ± 21	128 ± 18	567 ± 15	825 ± 34	864 ± 38	218 ± 31	292 ± 23	309 ± 23	579 ± 44	1006 ± 43	NV

Values are the percent of controls rats receiving filtered air and vehicle without 1-NN (average ± std (n=4))
Not Valid (NV), samples were out of quantitative range high
Grey background are statistically higher than their corresponding dosed filter air treated rats
